# The Clinical and Economic Impact of Employees Who Are Care Partners of Patients with Multiple Sclerosis by Disease Severity

**DOI:** 10.36469/001c.57593

**Published:** 2023-04-13

**Authors:** Barry Hendin, Richard A. Brook, Ian A. Beren, Nathan Kleinman, Cindy Fink, Amy L. Phillips, Carroline Lobo

**Affiliations:** 1 University of Arizona, Tucson; 2 Better Health Worldwide, Inc, Newfoundland, New Jersey; 3 Workpartners LLC, Cheyenne, Wyoming; 4 Care Partner Advisor; 5 EMD Serono, Rockland, Massachusetts

**Keywords:** multiple sclerosis, employee, care partner, severity, cost

## Abstract

**Background:** Research on employee care partners of patients with multiple sclerosis (MS) is limited. **Objectives:** The clinical and economic impact on employee care partners was evaluated by MS disease severity. **Methods:** Employees with spouses/domestic partners with MS from the Workpartners database (Jan. 1, 2010–Dec. 31, 2019) were eligible if: spouse/partner had at least 3 MS-related (ICD-9-CM/ICD-10-CM:340.xx/G35) inpatient/outpatient/disease-modifying therapy claims within 1 year (latest claim = index date); 6-month pre-index/1-year post-index enrollment; and age 18 to 64 years. Employee care partners’ demographic/clinical characteristics and direct/indirect costs were compared across predetermined MS severity categories. Logistic and generalized linear regression modeled the costs. **Results:** Among 1041 employee care partners of patients with MS, 358 (34.4%) patients had mild MS, 491 (47.2%) moderate, and 192 (18.4%) severe. Mean (standard error [SE]) employee care partner age was 49.0 (0.5) for patients with mild disease, 50.5 (0.4) for moderate, 51.7 (0.6) for severe; percent female care partners was 24.6% [2.3%] mild, 19.8% [1.8%] moderate, 27.6% [3.2%] severe; and mean care partner Charlson Comorbidity Index scores 0.28 (0.05) mild, 0.30 (0.04) moderate, 0.27 (0.06) severe. More care partners of patients with moderate/severe vs mild MS had hyperlipidemia (32.6%/31.8% vs 21.2%), hypertension (29.5%/29.7% vs 19.3%), gastrointestinal disease (20.8%/22.9% vs 13.1%), depression (9.2%/10.9% vs 3.9%), and anxiety 10.6%/8.9% vs 4.2%). Adjusted mean medical costs were greater for employee care partners of patients with moderate vs mild/severe disease (*P*<.001). Pharmacy costs (SE) were lower for employee care partners of mild vs severe/moderate patients (*P*<.005). Sick leave costs (SE) were greater for employee care partners of mild/severe vs moderate patients (*P*<.05). **Discussion:** Employee care partners of patients with moderate/severe vs mild MS had more comorbidities (ie, hypertension, gastrointestinal disease, depression, and anxiety) and higher pharmacy costs. Employee care partners of patients with moderate vs mild/severe MS had higher medical and lower sick leave costs. Treatment strategies that improve patient outcomes may reduce employee care partner burden and lower costs for employers in some instances. **Conclusions:** Comorbidities and direct/indirect costs of employees whose spouses/partners have MS were considerable and varied with MS severity.

## BACKGROUND

Multiple sclerosis (MS) is a chronic, recurrent, inflammatory, demyelinating disorder of the central nervous system.^1^ Although the clinical course of MS is highly variable, disease pathology in MS is typically continuous, even during periods of apparent remission, and usually leads to permanent disability.^2^ The clinical, economic, and humanistic burden of MS is substantial as it is a chronic, unpredictable, and progressive condition.^3^ The total economic burden of MS in the United States is estimated to be $85.4 billion, with $63.3 billion in direct medical costs and $22.1 billion in indirect and nonmedical costs.^4^ Multiple sclerosis typically affects patients at a young age, resulting in a greater loss of productivity and quality of life (QOL).^5^

Multiple sclerosis not only affects the patient’s productivity and QOL but also their care partner’s productivity and QOL.^6^ Multiple sclerosis is a chronic disease requiring long-term care. It is estimated that 80% of the care for patients with MS is provided by informal unpaid care partners,^7^ and care partners of patients with MS spend an average of 6.5 hours a day dedicated to caring tasks.^8^ Such responsibilities clearly have implications for other domains of life, such as employment.^9^ Multiple sclerosis care partners’ experience is highly variable and may differ on the basis of the care recipient, the care partner, and contextual factors.^9–12^ Caring for a patient with MS can negatively influence care partner physical and mental health, as well as impact their financial situation.^13^ Compared with non-care partners, MS care partners have been shown to have significantly greater activity impairment; poorer mental and physical QOL and health utility scores; and more healthcare provider, emergency department, and hospital visits.^9,10,12–15^

Although previous studies have evaluated the burden among care partners of patients with MS, there has been very limited research with objective data on employee care partners of patients with MS. Primary data regarding the indirect costs of MS are not readily available and are not commonly evaluated. Employers, healthcare providers, and care partners will benefit from an improved understanding of the impact of MS on care partners. Hence, the objectives of this study were to evaluate the 1-year clinical and economic impact on employee care partners of patients with MS stratified by MS patient disease severity.

## METHODS

### Data Source

Workpartners (formerly known as Human Capital Management Services [HCMS], which was acquired by Workpartners in 2017) is a health benefits consultant for a number of large US employers with diverse salary, job type, employee age, sex, and geographic region demographics. The employers are in a variety of industries, including manufacturing, insurance, retail, transportation, telecommunications, healthcare, grocery, and pharmaceuticals. Employers consented to have their data analyzed to learn how to improve health management for their employee populations.

The Workpartners Research Reference Database (RRDb) currently includes approximately 2.8 million employees (and insured spouses/partners) who were employed during a time between January 1, 2001, and June 30, 2019. This nontransferable database comprises healthcare claims, inpatient utilization, and pharmaceutical expenditures for the employees and their eligible dependents. The RRDb contains additional information not found in traditional expenditure databases such as information on employees’ short- and long-term disability claims, workers’ compensation claims, and sick leave claims. The RRDb also contains employee-specific information on demographics, company type, job type, employment status, salary, and health plan. Prior to research use, the data were de-identified to comply with HIPAA (Health Insurance Portability and Accountability Act) and contractual obligations with the employer contributors. Ethics approval from an institutional review board and informed consent were not required given the use of de-identified data and HIPAA compliance.

### Study Population

To be included in the study, employees were required to have spouses/domestic partners who were 18 to 64 years of age and had at least 3 MS-related (*International Classification of Diseases, Ninth Revision, Clinical Modification* [ICD-9-CM] code: 340.xx and ICD-10-CM code: G35) inpatient, outpatient, or MS disease-modifying therapy (DMT) claims within a 1-year period (latest claim with ≥12 months follow-up = index date) from July 1, 2010, to June 30, 2019. The employees and their spouses/partners were required to have continuous eligibility for at least 6 months before the index date (ie, eligible to receive healthcare benefits during the 6-month time period prior to index date; baseline period) and 12 months after the index date (follow-up period). Any employees with a diagnosis of MS were excluded. Disease modifying therapies considered for inclusion were subcutaneous interferon beta-1a, intramuscular interferon beta-1a, peginterferon beta-1a, subcutaneous interferon beta-1b, glatiramer acetate, daclizumab, teriflunomide, fingolimod, dimethyl fumarate, alemtuzumab, mitoxantrone, ocrelizumab, and natalizumab. Patients using natalizumab who also had Crohn’s disease or irritable bowel diagnosis were excluded.

### Disease Severity Categorization

Published literature informed a mapping exercise with a clinical expert to identify MS-related symptoms indicating a high likelihood of having severe, moderate, or mild disease (mutually exclusive stepwise categories) using diagnostic/medication/healthcare procedural codes.^16^ Patients were categorized as having mild, moderate, or severe disease based on a combination of the post-index symptoms within 4 body systems: bladder/bowel (eg, urinary/stool incontinence, visit to urologist, etc.), psychiatric (anxiety/depression), cognitive (dementia/cognitive impairment), and physical function (spasticity, wheelchair/cane/walker use, etc.). A more detailed description of disease severity can be found in the **Supplementary Appendix**.

### Baseline Demographic Characteristics

Baseline demographic characteristics of employees and spouses/partners with MS were assessed during the 6 months prior to index DMT initiation and were compared across MS disease severity categories. Demographic characteristics that were evaluated included age at index (continuous), sex, race, region (based on first-digit zip codes), tenure of employment, salary, exempt/nonexempt status, and full-time/part-time status.

### Clinical Characteristics

Clinical characteristics included: overall comorbidity as measured by the Charlson Comorbidity Index (CCI) and individual rates of hyperlipidemia, hypertension, gastrointestinal disease, depression, thyroid disease, anxiety, arthritis, chronic lung disease, diabetes, alcohol abuse diagnosis, suicide ideation/attempt, chronic pain syndrome, substance abuse, opioid abuse, and tobacco use.

### Direct Costs, Indirect Costs, and Absence Days

Direct costs were assessed during 12-month follow-up and were compared across MS disease severity categories. Direct costs included medical and drug costs. Medical costs were calculated based on claims filed under the medical benefit including inpatient hospitalizations, outpatient hospital or clinic visits, emergency department visits, office visits, laboratory tests and procedures, and other. Drug costs were calculated based on claims filed under the prescription benefit. Measures of indirect costs that were evaluated and compared included sick leave, short-term disability, long-term disability, and workers’ compensation. Absence days were obtained from employee payroll records and disability/workers’ compensation claims data and were not imputed from medical claims data. Indirect costs for absence days were obtained from actual employee cost data.

### Analyses

All study variables were analyzed descriptively. Categorical and binary variables were summarized using frequencies and percentages. Continuous variables were summarized using means and standard errors. Baseline demographics, clinical characteristics, follow-up comorbidities, and direct and indirect costs were compared across patient disease severity categories. Chi-square tests evaluated differences between categorical variables, and *t*-tests evaluated differences in continuous variables. A *P* value of <.05 was used to determine statistical significance. This study was exploratory in nature, and *P* values were not adjusted for multiplicity.

Cost and lost time data are highly non-normal, have many zero values, and are highly skewed. To get the most accurate comparison of these outcomes, *t*-tests on matched samples are generally not adequate, and 2-stage regression models are preferred to account for the unusual distributions of the outcomes.

Each outcome was modeled separately. The first stage used logistic regression to model the likelihood of an outcome greater than zero (eg, those with a disability claim vs those without). The second stage used generalized linear models (GLM) with a gamma distribution and log link on the portion of the population with a greater-than-zero outcome. The results of the GLM were then combined with the results of the logistic model to reach an expected value of cost or days for all employees. To adjust for confounding, independent variables were evaluated for inclusion in the models by the stepwise selection process and included age (as of the index date), tenure of employment (as of the index date), sex, marital status, salary, exempt/non-exempt status, full-time/part-time status, race, CCI score, and region of the country. Costs were adjusted to 2019 dollars (the year of the end of the data collection) using components of the Consumer Price Index from the US Bureau of Labor Statistics.

Employee care partners with missing baseline characteristic data were included in the analyses. Analysis of each medical leave cost or lost time (days absent) variable, however, included only those employee care partners that were continuously eligible and/or enrolled in the appropriate medical leave plan for the entire study period. This ensures that employees with missing cost or lost time information, due to ineligibility for a benefit, did not skew the results for that benefit. Statistical analyses were performed using SAS version 9.4 (SAS Institute, Inc, Cary, North Carolina).

## RESULTS

A total of 353 802 employees in the database had spouses/partners; 1179 of these employees had a spouse/partner who met the criteria for a diagnosis of MS, and 1041 of the employee and spouse/partner pairs had continuous eligibility for the study and baseline periods and met all the study eligibility criteria. Among the 1041 eligible care employee and spouse/partner pairs, 358 (34.4%) of the patients were classified with mild, 491 with moderate (47.2%), and 192 with severe MS (18.4%).

### Baseline Demographic and Clinical Characteristics

Baseline demographic and clinical characteristics of patients with MS and employee care partners are presented in **Table 1**. Patient and employee care partner age increased with increasing disease severity category. Mean (standard error [SE]) patient age was lower for mild (47.9 [0.5]) vs moderate (49.7 [0.4]) and vs severe (50.7 [0.7]) patients. Mean (SE) employee care partner age was also lower for mild (49.0 [0.5]) vs moderate (50.5 [0.4]) and vs severe (51.7 [0.6]) patients. The only significant sex difference was a higher proportion of female employee care partners of patients with MS in the severe cohort (27.6% [3.2%]) compared with the moderate cohort (19.8% [1.8%]).

**Table 1.** Baseline Demographic and Clinical Characteristics of Patients With Multiple Sclerosis and Employee Care Partners

	**Mild (n=358)**		**Moderate (n = 491)**		**Severe (n = 192)**	
	**Mean**	**SE**	**Mean**	**SE**	**Mean**	**SE**
Patients with MS						
Age, y	**47.9** ^a,b^	0.51	**49.7** ^a^	0.41	**50.7** ^b^	0.66
Sex (% female)	76.3	2.3	80.2	1.8	73.4	3.2
Charlson Comorbidity Index (CCI) score	**0.34** ^a,b^	0.04	**0.55** ^a^	0.06	**0.74** ^b^	0.10
Care partners (employees)						
Age, y (at index date)	**49.0** ^a,b^	0.50	**50.5** ^a^	0.41	**51.7** ^b^	0.64
Sex (% female)	24.6	2.3	**19.8** ^c^	1.8	**27.6** ^c^	3.2
Race, %						
White	25.7	2.3	31.8	2.1	24.5	3.1
Black	3.1	0.9	2.2	0.7	2.6	1.2
Hispanic	**5.6** ^a^	1.2	**2.9** ^a^	0.8	4.2	1.4
Other race	1.7	0.7	1.0	0.5	0.0	0.0
Missing	64.0	2.5	62.1	2.2	68.8	3.4
Region by first digit of zip code, %d						
0	**9.5** ^a^	1.6	**5.5** ^a^	1.0	5.7	1.7
1	**10.6** ^b^	1.6	**9.4** ^c^	1.3	**17.7** ^b,c^	2.8
2	**6.7** ^a^	1.3	**11.2** ^a^	1.4	7.3	1.9
3	8.1	1.4	10.4	1.4	8.9	2.1
4	6.7	1.3	6.7	1.1	8.3	2.0
5	**9.0** ^b^	1.5	7.7	1.2	**4.2** ^b^	1.4
6	6.4	1.3	7.5	1.2	9.4	2.1
7	14.3	1.9	14.7	1.6	14.1	2.5
8	13.7	1.8	14.3	1.6	9.9	2.2
9	14.8	1.9	12.6	1.5	14.6	2.6
Job-related variables						
Annual salary, $	110 249	12 597	91 816	3457	80 735	4586
Full-time employment, %	79.6	2.1	82.9	1.7	83.9	2.7
Exempt, %	42.2	2.6	43.2	2.2	41.7	3.6
Employee tenure, y	11.1	0.52	11.4	0.43	11.8	0.73
CCI score	0.28	0.05	0.30	0.04	0.27	0.06

Bolded values indicate statistically significant differences between groups.

Abbreviations: CCI, Charlson Comorbidity Index; MS, multiple sclerosis; SE, standard error.

^a^*P* < .05 mild vs moderate.

^b^*P* < .05 mild vs severe.

^c^*P* < .05 moderate vs severe.

^d^Zip codes are numbered with the first digit representing certain US states: 0 = Connecticut, Massachusetts, Maine, New Hampshire, New Jersey, Puerto Rico, Rhode Island, Vermont, and Virgin Islands; 1 =Delaware, New York, and Pennsylvania; 2 = District of Columbia, Maryland, North Carolina, South Carolina, Virginia, and West Virginia; 3 = Alabama, Florida, Georgia, Mississippi, and Tennessee; 4 = Indiana, Kentucky, Michigan, and Ohio; 5 = Iowa, Minnesota, Montana, North Dakota, South Dakota, and Wisconsin; 6 = Illinois, Kansas, Missouri, and Nebraska; 7 = Arkansas, Louisiana, Oklahoma, and Texas; 8 = Arizona, Colorado, Idaho, New Mexico, Nevada, Utah, and Wyoming.

Employee care partner racial/ethnic information, job-related information, and region of the country based on the zip code for the employees is also presented in **Table 1**. A larger proportion of employee care partners of patients with mild MS were Hispanic (5.6%) compared with employee care partners of patients with moderate MS (2.9%). No differences in job-related variables were observed across MS disease severity categories. Regional differences across the United States were identified for employee and spouse pairs living in zip codes that begin with 0, 1, 2, and 5. A greater proportion of employee care partners of patients with mild vs moderate MS resided in the Connecticut, Massachusetts, Maine, New Hampshire, New Jersey, Puerto Rico, Rhode Island, Vermont, and Virgin Islands regions (9.5% vs 5.5%). A greater proportion of employee care partners of patients with severe vs mild (17.7% vs 10.6%) and vs moderate (9.4%) MS resided in Delaware, New York, and Pennsylvania. A greater proportion of employee care partners of patients with moderate vs mild MS resided in District of Columbia, Maryland, North Carolina, South Carolina, Virginia, and West Virginia (11.2% vs 6.7%). A greater proportion of employee care partners of patients with mild vs severe MS resided in Iowa, Minnesota, Montana, North Dakota, South Dakota, and Wisconsin (9.0% vs 4.2%).

Patients with MS in the mild disease severity category had lower mean (SE) baseline CCI scores (0.34 [0.04]) than patients in the moderate (0.55 [0.06]) or severe (0.74 [0.10]) disease severity categories (**Table 1**). There were no differences in baseline CCI scores for employee care partners when stratified by patient MS disease severity category (**Table 1**).

### Care Partner Clinical Comorbidities During Follow-up

**Figure 1.**
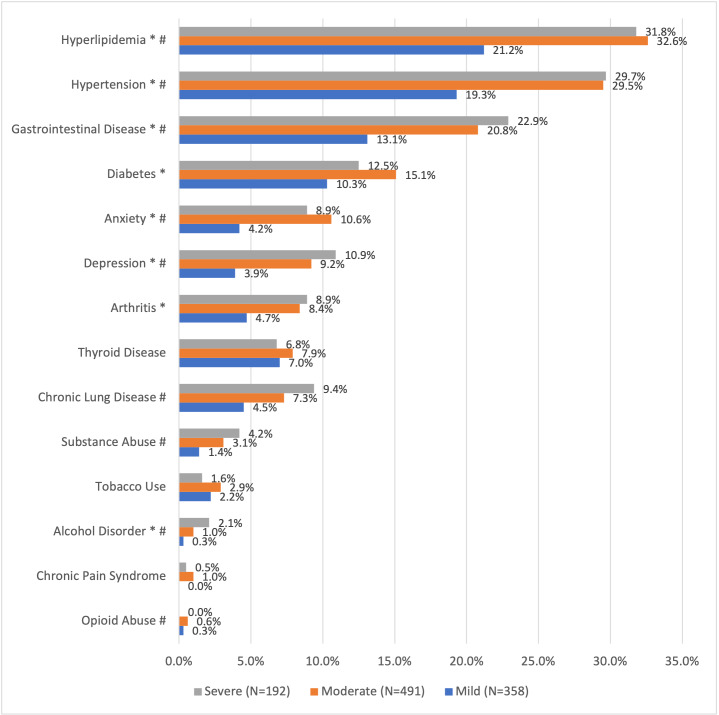
Comorbidities During 1-Year Follow-up for Employee Care Partners by Spouse/Partners’ Multiple Sclerosis Disease Severity Category Abbreviation: MS, multiple sclerosis. *P < .05 mild vs moderate. #*P* < .05 mild vs severe.

A greater proportion of employee care partners of patients with moderate and severe vs mild MS had hyperlipidemia (32.6% and 31.8% vs 21.2%, respectively), hypertension (29.5% and 29.7% vs 19.3%, respectively), gastrointestinal disease (20.8% and 22.9% vs 13.1%, respectively), depression (9.2% and 10.9% vs 3.9%, respectively), and anxiety (10.6% and 8.9% vs 4.2%, respectively) (**Figure 1**). A greater proportion of employee care partners of patients with moderate vs mild MS had diabetes (15.1% vs 10.3%) and arthritis (8.4% vs 4.7%). A greater proportion of employee care partners of patients with severe vs mild MS had chronic lung disease (9.4% vs 4.5%), substance abuse (4.2% vs 1.4%), and alcohol disorder (2.1% vs 0.3%) (**Figure 1**).

Mean CCI scores during follow-up for employee care partners did not differ by patient MS disease severity category (mild 0.32 [0.05]; moderate 0.42 [0.04]; severe 0.41 [0.07]).

### Care Partner Costs

**Figure 2.**
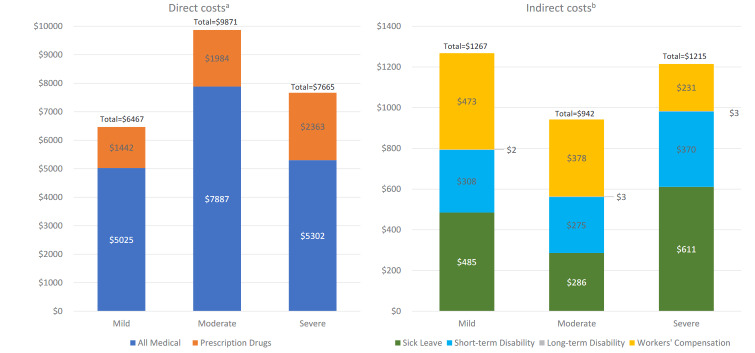
Adjusted 1-Year Mean Direct/Indirect Costs for Employee Care Partners by Spouse/Partners’ MS Disease Severity Category Due to rounding, some totals may not correspond with the sum of the separate costs. Abbreviation: MS, multiple sclerosis. ^a^Mean medical costs were greater for partners of moderate vs mild/severe patients (both *P* < .001) and mean pharmacy costs were lower for partners of mild vs severe/moderate patients (both *P* < .005). ^b^Mean sick leave costs were greater for partners of mild/severe vs moderate patients (both *P* < .05).

**Table 2** presents the employee care partner direct costs by patient MS disease severity category. Adjusted mean (SE) direct medical costs were greater for employee care partners of patients with moderate ($7887 [$518]) vs mild ($5025 [$390]) and severe ($5302 [$561]) MS. Pharmacy costs (SE) were lower for employee care partners of patients with mild ($1442 [$126]) vs severe ($2363 [$278]) and moderate ($1984 [$145]) MS (**Figure 2**).

**Table 2.** Adjusted Direct Costs During 1-Year Follow-up for Employee Care Partners by Disease Severity Category of Spouse/Partner With Multiple Sclerosis

**Cost Category^a^**	**Severe (1)**			**Moderate (2)**			**Mild (3)**			**Severe vs Mild**		**Moderate vs Mild**		**Severe vs Moderate**	
	**n**	**Adjusted Mean Cost**	**SE**	**n**	**Adjusted Mean Cost**	**SE**	**n**	**Adjusted Mean Cost**	**SE**	**Δ 1-3**	***P* Value (1-3)**	**Δ 2-3**	***P* Value (2-3)**	**Δ 1-2**	***P* Value (1-2)**
Care partner (employee), study period: Direct costs															
Medical costs	192	$5302	$561	491	$7887	$518	358	$5025	$390	$278	.6842	$2862	**.0000**	-$2585	**.0007**
Drug costs	192	$2363	$278	491	$1984	$145	358	$1442	$126	$921	**.0026**	$542	**.0048**	$379	.2275
Total direct costs^b^		$7665			$9871			$6467		$1199		$3405		-$2206	
Care partner (employee), study period: Likelihood of having direct costs															
Likelihood of having medical costs	192	92%	2.00%	491	94%	1.10%	358	91%	1.50%	0.60%	.8230	2.70%	.1435	-2.20%	.3425
Likelihood of having drug costs	192	90%	2.10%	491	92%	1.20%	358	86%	1.80%	4.40%	.1214	6.10%	**.0054**	-1.80%	.4726
Care partner (employee), study period: Direct costs (given costs >$0)															
Medical costs	170	$5789	$636	446	$8412	$571	308	$5520	$451	$269	.7298	$2892	**.0001**	-$2623	**.0022**
Drug costs	165	$2618	$326	430	$2156	$166	280	$1679	$160	$939	**.0097**	$477	**.0388**	$462	.2062

Abbreviations: Δ, difference between cohorts (means); MS, multiple sclerosis; SE, standard error.

^a^Costs were calculated using 2-stage (logistic-GLM) regression modeling by controlling for age, tenure, sex, race, exempt status, full-time/part-time status, salary, location, and Charlson Comorbidity Index Score (of the person being modeled).

^b^Because the direct costs components are highly significant and in the same direction, the total is likely to be highly significant given the consistent direction and *P* values for the components.

**Table 3** presents the employee care partner indirect costs by patient disease severity category. Sick leave costs (SE) were lower for employee care partners of patients with moderate ($286 [$45]) compared with mild ($485 [$76]) and severe ($611 [$130]) MS. There were no significant differences in short-term disability, long-term disability, and workers’ compensation costs across the 3 groups (**Figure 2**).

**Table 3.** Adjusted Indirect Costs During 1-Year Follow-Up For Employee Care Partners by Disease Severity Category of Spouse/Partner With Multiple Sclerosis

	**Severe (1)**			**Moderate (2)**			**Mild (3)**			**Severe vs Mild**		**Moderate vs Mild**		**Severe vs Moderate**	
**Cost Category^a^**	**n**	**Adjusted Mean Cost**	**SE**	**n**	**Adjusted Mean Cost**	**SE**	**n**	**Adjusted Mean Cost**	**SE**	**Δ 1-3**	***P* Value (1-3)**	**Δ 2-3**	***P* Value (2-3)**	**Δ 1-2**	***P* Value (1-2)**
Care partner (employee), study period: Indirect costs															
Absence costs (sick leave)	59	$611	$130	160	$286	$45	107	$485	$76	$126	.4028	−$198	.0248	$324	.0185
Short term disability costs	136	$370	$147	327	$275	$61	216	$308	$109	$62	.7359	−$33	.7913	$95	.5519
Long term disability costs	94	$3	$6	281	$3	$6	178	$2	$3	$2	.8077	$1	.8863	$1	.9399
Workers’ compensation costs	150	$231	$110	388	$378	$112	271	$473	$187	−$242	.2658	−$95	.6648	−$147	.3486
Care partner (employee), study period: Likelihood of having indirect costs															
Likelihood of having absence (sick leave) costs	59	33%	6.10%	160	23%	3.30%	107	34%	4.60%	−0.60%	.9381	−10.70%	.0586	10.10%	.1475
Likelihood of having short- term disability costs	136	5%	1.80%	327	6%	1.30%	216	4%	1.30%	0.90%	.6734	2.40%	.1984	−1.40%	.5204
Likelihood of having long-term disability costs	94	0%	0.60%	281	0%	0.20%	178	0%	0.30%	0.10%	.8313	0.10%	.7343	0.30%	.6636
Likelihood of having workers’ compensation costs	150	3%	1.40%	388	3%	0.90%	271	2%	0.90%	0.60%	.7200	0.60%	.6205	0.00%	.9853
Care partner (employee), study period: Indirect costs (given costs >$0)															
Absence (sick leave) costs	25	$1845	$299	55	$1245	$136	45	$1438	$174	$406	.2395	−$194	.3794	$600	.0674
Short-term disability costs	9	$7997	$2363	27	$4541	$775	12	$8347	$2136	−$350	.9124	−$3806	.0939	$3456	.1646
Long-term disability costs	2	$974	$110	2	$3820	$432	2	$887	$100	$88	.5562	$2934	<.0001	-$2846	<.0001
Workers’ compensation costs	7	$7600	$4471	17	$12 333	$4655	9	$19 438	$10 083	-$11 838	.2832	−$7105	.5223	-$4732	<.0001

Abbreviations: Δ, difference between cohorts (means); MS, multiple sclerosis; SE, standard error.

^a^Costs were calculated using 2-stage (logistic-GLM) regression modeling by controlling for age, tenure, sex, race, exempt status, full-time/part-time status, salary, location, Charlson Comorbidity Index Score (of the person being modeled).

**Table 4** presents the employee care partner absence days by patient disease severity category. Sick leave days were lower for employee care partners of patients with moderate (1.21 [0.19]) compared with mild (2.18 [0.25]] and severe (2.88 [0.61]) MS. There were no significant differences in absence days related to short-term disability, long-term disability, and workers’ compensation across the 3 groups.

**Table 4.** Adjusted Absence Days During 1-Year Follow-up for Employee Care Partners by Disease Severity Category of Spouse/Partner With Multiple Sclerosis

**Absence Days by Type**	**Severe (1)**			**Moderate (2)**			**Mild (3)**			**Comparison**					
	**n**	**Adjusted Mean Days**	**SE**	**n**	**Adjusted Mean Days**	**SE**	**n**	**Adjusted Mean Days**	**SE**	**Δ 1-3**	***P* Value (1-3)**	**Δ 2-3**	***P* Value (2-3)**	**Δ 1-2**	***P* Value (1-2)**
Care partner (employee), study period — Days															
Absence days (sick leave)	59	2.88	0.61	160	1.21	.19	107	2.18	0.35	0.70	.3139	−0.97	.0134	1.68	.0083
Short-term disability days	136	3.03	1.03	327	1.81	0.38	216	2.99	0.98	0.04	.9761	−1.18	.2646	1.22	.2678
Long-term disability days	94	0.91	1.45	281	0.21	0.28	178	1.05	1.20	−0.14	.9388	−0.84	.4968	0.69	.6377
Workers’ compensation days	150	1.01	1.21	388	1.13	0.54	271	0.10	0.06	0.91	.4522	1.02	.0578	−0.11	.9311
Care partner (employee), study period — Likelihood of having absence days															
Likelihood of absence (sick leave) days	59	33.4%	6.1%	160	22.9%	3.3%	107	32.7%	4.5%	0.7%	.9250	−9.8%	.0817	10.5%	.1324
Likelihood of short-term disability days	136	6.2%	2.1%	327	6.7%	1.4%	216	4.2%	1.4%	2.0%	.4251	2.5%	.2011	−0.5%	.8377
Likelihood of long-term disability days	94	0.4%	0.7%	281	0.2%	0.3%	178	0.4%	0.5%	0.0%	.9885	−0.2%	.6936	0.2%	.7712
Likelihood of workers’ compensation days	150	0.5%	0.6%	388	1.1%	0.5%	271	1.1%	0.6%	−0.6%	.4447	0.0%	.9886	−0.7%	.3938
Care partner (employee), study period — Days (given days >0)															
Absence (sick leave) days	25	8.63	1.36	54	5.27	0.56	44	6.67	0.79	1.96	.2126	−1.40	.1486	3.36	.0222
Short-term disability days	12	49.14	10.62	29	27.14	3.77	13	71.22	14.78	−22.07	.2252	−44.08	.0039	22.01	.0508
Long-term disability days	1	217.92	33.09	2	102.80	11.04	2	245.60	26.37	−27.68	.5130	−142.80	<.0001	115.12	.0010
Workers’ compensation days	1	217.60	4.98	6	100.29	0.94	4	9.18	0.10	208.42	<.0001	91.11	<.0001	117.31	<.0001

Abbreviations: Δ, difference between cohorts (means or percentages); MS, multiple sclerosis; SE, standard error.

Days were calculated using 2-stage (logistic-GLM) regression modeling by controlling for age, tenure, race, exempt status, full-time/part-time status, salary, location, Charlson Comorbidity Index Score (of the person being modeled), and other measures.

## DISCUSSION

The long course and duration of MS frequently requires family members or other care partners to perform multiple roles of caregiving and of assuming the financial and household responsibilities.^17^ However, there has been limited research on employee care partners of patients with MS to date. This real-world study of US employer data sought to quantify the clinical and economic burden of employee care partners of patients with MS.

Study findings showed that employees with spouses/partners with severe or moderate MS had significant comorbidities compared with spouses/partners with mild MS. More care partners of patients with moderate/severe vs mild MS had hyperlipidemia (32.6%/31.8% vs 21.2%), hypertension (29.5%/29.7% vs 19.3%), gastrointestinal disease (20.8%/22.9% vs 13.1%), depression (9.2%/10.9% vs 3.9%), and anxiety (10.6%/8.9% vs 4.2%). These findings are consistent with those of McKenzie et al,^18^ who analyzed survey data from the NARCOMS (North American Research Committee on Multiple Sclerosis) Registry and found that care partners of people with MS had substantial physical and psychological health concerns. Use of medications for specific health concerns was common among care partners, with the most frequent concerns being hypertension (28.5%), hypercholesterolemia (26.3%), mood disorders (17.8%), stress/anxiety (14.0%), headache (13.4%), and sleep disturbance (12.9%).^18^ The authors found that burden was greater for patients with primary progressive MS and secondary progressive MS than for relapsing remitting MS (both *P*<.0001) and for primary progressive MS than for secondary progressive MS (*P* = .002).^18^

Caring for a loved one with MS can often be fulfilling^12,19^; however, it can also become overwhelming, physically and emotionally challenging, and isolating.^9,12,17^ The chronic stress of caring for a partner may be a risk factor for developing chronic conditions and for engaging in unhealthy behaviors such as sedentary lifestyle, poor nutrition, social isolation, and use of substances such as alcohol or prescription drugs.^20,21^ It is essential to understand the importance of the employee care partner and the care partner’s wellness in the management of a chronic neurological condition such as MS.^17^ To be able to care for their loved one, employee care partners must take care of themselves both physically and emotionally.

Findings from the current study also showed that direct and indirect costs varied with MS severity. Employee care partners of patients with moderate/severe vs mild MS had higher pharmacy costs; however, employee care partners of patients with moderate vs mild/severe MS had higher medical costs and lower sick leave costs. It is unclear why employee care partners of patients with moderate MS had higher medical costs compared with care partners of patients with severe MS. One possible explanation is that employee care partners of patients with severe MS may not have time available to seek medical care as the demands for the care recipient increase physically, emotionally, and cognitively.^17^ In these cases, employee care partner health outcomes would be worsening; however, they would not be obtaining adequate medical care. It is also possible that patients with severe MS qualify for more direct medical resources compared with those with moderate disease and receive more external support, hence shifting some of the burden away from the employee care partners. The additional medical resources and more external support may also explain the lower sick leave costs for employee care partners of patients with moderate vs mild MS. If managing their own physical and psychological medical conditions is a low priority for care partners, they may be less likely to participate in essential wellness activities and their burden and problems will likely be compounded,^17^ as the experience of comorbidities can make caring more difficult.^22^

The development and availability of a wide range of DMTs today enable patients with MS to better manage their condition relative to previous decades.^9^ Disease-modifying treatment strategies that improve patient outcomes by reducing relapses and disability progression may result in less clinical and humanistic burden for care partners and lower costs for employers.^23,24^ A recent study of patients in Denmark demonstrated that a clinically stable disease course was associated with a reduced risk of patients losing income from salaries and disability pension compared with those that did not have a clinically stable disease course.^25^ Adherence to DMT treatment regimens is necessary for optimal management of patients with MS, as DMT nonadherence has been shown to be related to the risk of relapses and increased healthcare costs.^26,27^ Studies in other therapeutic areas have shown that simpler and less frequent DMT dosing produces greater patient adherence than more frequent administration.^28–30^

The current study reinforces that MS is a chronic and debilitating disease that poses a substantial employer burden in terms of medical, absenteeism, and disability costs. Differences in cost between employees with MS vs non-MS controls and between treated and untreated employees with MS were previously reported in a study by Ivanova et al,^31^ which showed that employees with MS had significantly higher rates of physical, mental health, and other neurological disorders, and more than 4 times greater indirect costs compared with employee controls. Previous studies have also demonstrated that the use of DMTs in employees with MS is associated with lower rates of relapses and substantial medical and indirect cost savings.^23,31–33^

Strengths of the current study include its uniqueness in evaluating the clinical and economic impact of employee care partners of patients with MS and its presentation of primary data regarding the indirect costs of MS, which are not readily available and are not commonly evaluated. The study was completed in a unique US database that included direct and indirect costs and absences based on 4 absence benefit types.

There are several limitations of the current study related to the use of administrative claims data. The ICD-9-CM and ICD-10-CM codes for MS do not distinguish between different disease courses, such as relapsing-remitting, secondary progressive, and primary progressive. Potential limitations inherent to the use of administrative data include the risk of clerical inaccuracies, recording bias secondary to financial incentives, and temporal changes in billing codes. The analysis was restricted to data available in a health claims database. The findings do not reflect the full burden of care partners of patients with MS as the claims data only reflect interactions with the healthcare system and short- and long-term disability claims, workers’ compensation claims, and sick leave claims. Furthermore, other unmeasured factors may have confounded the observed relationships (eg, clinician-reported relapses and magnetic resonance imaging data). Future prospective studies could more comprehensively capture employee care partner burden and provide context for the resource utilization results observed in the current study. The algorithm used to assign care partners of patients with MS to varying severity levels used administrative claims data and may have underestimated the severity of the patients’ condition (see **Supplementary Appendix**). While the database confirmed that the care partners were spouses (or domestic partners) of the patients, there was no information in the database as to the existence or availability of other caregivers. It is unclear why some of the medical and sick leave cost results by MS severity were counterintuitive and in contrast to previous literature showing a positive correlation between patient severity and care partner time burden.^8^ These findings of higher medical and lower sick leave costs for employees who cared for less severe patients warrant further investigation to better understand the reasons and meet employees’ needs. The 6-month baseline period was selected to maximize the number of patients available for the analysis; however, a longer baseline period of up to 12 months would be preferred for future analyses as more patient data accumulate. The use of stepwise selection for the regression has limitations, including that stepwise regression may have incorrect results and inherent biases in the process itself. However, the stepwise procedure allowed us to force some independent variables into the models that were explored during the review process to ensure face validity. Because of the sample size of patients with MS available in the Workpartners RRDb, DMT was not considered as a variable in the model, as the population was not large enough to enable this granularity in the analysis. Finally, these administrative claims data were derived from US employee care partners and their spouses/partners with MS with commercial health insurance and therefore may not be generalizable to US employee care partners and their spouses/partners who do not have employer-based health insurance benefits. The findings also may not be generalizable to patients who live in other countries.

## CONCLUSIONS

This real-world, health claims database study showed that employee care partners with spouses/domestic partners with MS had significant comorbidities and direct and indirect costs that varied with MS severity. Employee care partners of patients with moderate/severe vs mild MS had more comorbidities (ie, hypertension, gastrointestinal disease, depression, and anxiety) and higher pharmacy costs. Employee care partners of patients with moderate vs mild/severe MS had higher medical costs and lower sick leave costs. Treatment strategies that improve patient outcomes by reducing relapses and disability progression may result in less clinical and humanistic burden for some employee care partners and lower costs for employers in some instances. The assessment of such strategies is an important area of future research.

### Author Contributions

B.H., A.L.P., and C.L. conceived and designed the study, acquired and interpreted the data, and drafted the work and revised it critically. R.A.B., I.A.B., and N.K. conceived and designed the study, analyzed and interpreted the data, and drafted the work and revised it critically. C.F. interpreted the data and revised the work critically.

### Ethical Approval of Studies

Ethics approval from an institutional review board and informed consent were not required as the data were from an anonymous, de-identified, administrative claims database compliant with HIPAA.

### Meeting Presentation

This study was presented as a poster at the Consortium of Multiple Sclerosis Centers (CMSC) Annual Meeting; October 25-28, 2021; Orlando, Florida.

### Data Availability

The data were obtained through a license agreement with Workpartners.

## Supplementary Material

Online Supplementary Material
