# Supplementary material for: The Clinical and Economic Impact of Employees Who Are Care Partners of Patients with Multiple Sclerosis by Disease Severity
Source: J Health Econ Outcomes Res. 2023 Apr 13;10(1):91–101. doi: 10.36469/001c.57593 (PMC10105615; doi:10.36469/001c.57593)
Supplement: Online Supplementary Material [file jheor_2023_10_1_57593_156375.pdf]

Journal of Health Economics and Outcomes Research

Online Supplementary Material

The Clinical and Economic Impact of Employees Who Are Care Partners of Patients with Multiple Sclerosis by Disease Severity. *JHEOR*. **2023;10(1):91-101.** doi:10.36469/jheor.2023.57593

Appendix: Assessment of Disease Severity

This supplementary material has been provided by the authors to give readers additional information about their work.

This is an open-access article distributed under the terms of the Creative Commons Attribution 4.0 International License (CCBY-4.0). View this license's legal deed at http://creativecommons.org/licenses/by/4.0 and legal code at http://creativecommons.org/licenses/by/4.0/legalcode for more information.

APPENDIX: ASSESSMENT OF DISEASE SEVERITY

Assessment of MS Severity

The claims for the patients with multiple sclerosis (MS) were examined to determine the disease severity. Based on clinical input from Dr. Hendin, four (4) body systems were examined.

Patients with MS were categorized as potentially:

- **Severe**: if they had ≥1 system category code from the table below
- **Moderate**: if they had no severe codes and any of the moderate codes from the table below
- **Mild**: if they had no severe or moderate codes

Multiple Sclerosis Severity Level Criteria

Body System	Potential Classification		
	**Severe: a patient with MS and indicators for ≥1 system category below[a]**	**Moderate: a patient with MS, no Severe codes, and any of the following Moderate codes[a]**	**Mild: a patient with MS and no Severe or Moderate codes[a]**
Bladder/bowel	Overactive bladder and/or visit to a urologist<br>Urinary incontinence AND Rx for urinary incontinence AND visit to a urologist<br>Stool incontinence	Urinary incontinence diagnosis AND Rx for urinary incontinence	Urinary incontinence
Psychiatric	(Depression OR Rx for antidepressants) OR (anxiety OR Rx for antianxiety medications) AND visit to a psychiatrist	(Depression OR Rx for antidepressants) OR (anxiety OR Rx for antianxiety medications)	
Cognitive	Dementia (non-Alzheimer's)	Mild cognitive impairment	
Physical function	Spasticity diagnosis AND Baclofen IV<br>Evidence of a wheelchair<br>Evidence of a fall<br>(Dysesthesia/paresthesia/hyperesthesia) AND pain medications/opioids and a visit to a pain specialist	Spasticity diagnosis AND (Botox administration codes)<br>(Dysesthesia/paresthesia/ hyperesthesia) AND pain-modulating medications or opioids<br>Evidence of cane/walker<br>Rx for dalfampridine (Ampyra)	

[a]Prescription codes, ICD-9/-10, and CPT codes were finalized in a workbook with the study team.

Note: Pain, including fibromyalgia, trigeminal neuralgia, other pain syndromes, numbness/tingling, fatigue, soft-tissue disorders, and optic neuritis were not included since these symptoms could be present across the 3 categories.
